# Aspiration-Assisted Catheter-Directed Foam Sclerotherapy Using the Sclerosafe Device for Anterior Saphenous Vein Insufficiency: A Retrospective Study

**DOI:** 10.3390/healthcare14172759

**Published:** 2026-09-01

**Authors:** Michał-Goran Stanišić, Krzysztof Czajkowski, Joanna Borecka-Sobczak, Szymon Markiewicz, Magdalena Snoch-Ziołkiewicz, Joanna Błaszak, Jolanta Tomczak

**Affiliations:** 1 Department of Vascular Surgery, Poznan University of Medical Sciences, 61-848 Poznań, Poland; 2 Angiodiabetica—Centre for Vascular Diseases and Diabetic Foot, 61-541 Poznań, Poland; 3 JBS Klinika—Specialist Medical Center of Phlebology Gdańsk, 80-252 Gdańsk, Poland; 4 General and Vascular Surgery Department, WSZ Elbląg, 82-400 Elbląg, Poland

**Keywords:** anterior saphenous vein, foam sclerotherapy, catheter-directed sclerotherapy, Sclerosafe, venous insufficiency, non-thermal ablation, varicose veins, polidocanol

## Abstract

**Highlights:**

**What are the main findings?**

- Aspiration-assisted catheter-directed foam sclerotherapy using a dedicated dual-lumen catheter (Sclerosafe) achieves a high technical success rate in the treatment of anterior saphenous vein insufficiency, enabling sonographically assessed displacement of intraluminal blood from the target segment during sclerosant delivery without tumescent anaesthesia.
- The technique is safe and was performed in a fully outpatient setting across a clinically relevant range of vessel diameters and segment lengths, with no major adverse events. Minor events were resolved spontaneously in all cases.

**What are the implications of the main finding?**

- The aspiration-assisted delivery mechanism is intended to address the principal limitation of conventional foam sclerotherapy—incomplete intraluminal blood displacement—and achieved a 12-month occlusion estimate of 90.9% without tumescent anaesthesia. In the absence of a control group, no comparison with thermal ablation is implied; the technique may nevertheless suit the superficial and variable anatomy of the ASV, where tumescent access is technically demanding.
- These hypothesis-generating results support the design of randomised or registry-based comparative trials to establish the role of aspiration-assisted CDFS within the evidence hierarchy for non-thermal, non-tumescent management of superficial venous insufficiency.

**Abstract:**

**Objective:** Anterior saphenous vein insufficiency is an underappreciated but clinically significant source of superficial venous reflux. Catheter-directed foam sclerotherapy represents a minimally invasive treatment approach, yet optimisation of sclerosant-to-endothelium contact remains a recognised technical challenge. The Sclerosafe system (VVT Medical, Kefar Sava, Israel)—a dual-lumen aspiration-assisted sclerotherapy device—was developed to address this limitation. The aim of this study was to evaluate the safety and efficacy of the Sclerosafe device in the treatment of anterior saphenous vein insufficiency. **Methods:** Retrospective analysis of prospectively collected data from 56 patients treated for isolated anterior saphenous vein insufficiency between 2020 and 2023. All procedures were performed in an outpatient setting under local anaesthesia (2 mL of 1% lignocaine at the puncture site) without tumescent infiltration. The Sclerosafe catheter was introduced directly, without an introducer sheath, through a single ultrasound-guided puncture of the ASV at the lowest point of reflux, and its tip was positioned 2 cm distal to the confluence with the common femoral vein. Treated segment length ranged from 8 to 17 cm (mean 10 cm) and vessel diameter from 4 to 12 mm (mean 6 mm). Polidocanol 2% foam (5 mL per session, Tessari technique) was administered and a standardised compression regimen was applied. Duplex ultrasound follow-up was conducted at 1, 2, 6, and 12 months; no venous severity, pain or quality-of-life score was recorded. **Results:** Technical success was achieved in all 56 cases. Complete occlusion of the treated segment was confirmed by duplex ultrasound at one month in 52 of 56 patients (92.9%). Occlusion rates at 6 months and 12 months were 92.5% (49/53 evaluable) and 90.4% (47/52 evaluable), with 3 and 4 cumulative patients lost to follow-up, respectively. Five occlusion failures were recorded in total: four primary failures at one month and one recanalisation between 6 and 12 months. Kaplan–Meier estimates of primary occlusion probability were 92.9% at 1 month (95% CI 86.1–99.6%), 92.9% at 6 months (no interval events), and 90.9% at 12 months (95% CI 83.3–98.5%). Minor adverse events included superficial skin pigmentation (8 patients, 14.3%), localised tenderness (6 patients, 10.7%), and transient bruising (11 patients, 19.6%); all resolved without specific intervention. No deep vein thrombosis, pulmonary embolism, or neurological complications were observed. **Conclusions:** Aspiration-assisted catheter-directed foam sclerotherapy using the Sclerosafe device demonstrated promising safety and anatomical efficacy for the treatment of anterior saphenous vein insufficiency, with a 12-month Kaplan–Meier occlusion estimate of 90.9%, without tumescent anaesthesia and in a fully outpatient setting. In the absence of a control group and clinical outcome measures, the technique cannot be presented as an established alternative to thermal or non-thermal ablation, but it may be a technically attractive option in patients with clinical CEAP class C2–C4 disease. Prospective comparative studies are warranted to confirm these findings.

## 1. Introduction

Lower limb varicose veins constitute a highly prevalent chronic venous condition, affecting an estimated 15–25% of the adult population with a well-documented female predominance [1]. Within this clinical spectrum, anterior saphenous vein (ASV) insufficiency is an underappreciated but clinically significant entity. The ASV—formerly termed the anterior accessory great saphenous vein and re-designated the anterior saphenous vein by a 2024 multisocietal consensus in recognition of its truncal anatomical characteristics [2,3]—runs along the anterolateral aspect of the thigh within its own saphenous compartment, lateral to the great saphenous vein (GSV), and may serve as an independent source of superficial reflux, either in isolation or in continuity with the GSV [2,3].

Current guidelines have fundamentally reshaped the therapeutic approach to superficial venous insufficiency. Both the ESVS 2022 Clinical Practice Guidelines and the NICE guidance recommend thermal endovenous modalities—endovenous laser ablation (EVLA) and radiofrequency ablation (RFA)—as first-line treatment for GSV reflux, based on robust clinical evidence [4]. These techniques, however, share a common operational requirement: perivenous tumescent anaesthesia and favourable vessel geometry, which may preclude their use in tortuous, small-calibre, or anatomically superficial venous segments, such as the ASV.

Within the category of procedures that dispense with both thermal energy and tumescent infiltration, three principal modalities have gained clinical adoption: mechanochemical ablation (MOCA), cyanoacrylate closure (CAC), and foam sclerotherapy in their various delivery configurations [5]. Of these, catheter-directed foam sclerotherapy (CDFS) has attracted particular interest due to its capacity to optimise sclerosant distribution. Positioning the delivery catheter within the target segment maximises blood displacement and prolongs sclerosant-to-endothelium contact—a significant determinant of obliteration efficacy. In a meta-analysis of 3689 patients, three-year venous occlusion rates of 82.4% for CDFS compared with 62.9% for conventional ultrasound-guided foam sclerotherapy (UGFS) have been reported [6].

Despite these advances, a recognised limitation of CDFS—as with all sclerotherapy techniques—is incomplete blood displacement from the treated lumen, which dilutes the sclerosant and reduces its endothelial contact time. The Sclerosafe system (VVT Medical, Kefar Sava, Israel) represents a rational modification of CDFS: a dual-lumen catheter that simultaneously aspirates blood from the treated segment while delivering the sclerosant, aiming to optimise conditions for vessel obliteration [7]. While initial reports have addressed its application in the GSV and small saphenous vein (SSV) insufficiency, data concerning the ASV remain limited.

The aim of this study is to report the clinical outcomes of aspiration-assisted catheter-directed foam sclerotherapy using the Sclerosafe device in a consecutive series of patients with isolated ASV insufficiency and to contextualise these findings within the current evidence base for catheter-directed foam sclerotherapy.

## 2. Methods

### 2.1. Study Design and Patient Selection

This is a retrospective analysis of prospectively collected data from a single-centre cohort treated between January 2020 and December 2023. Data were gathered prospectively as part of the institutional standard of care protocol for all patients undergoing endovenous interventions; the retrospective analytical approach was conducted in accordance with institutional data governance standards, which do not mandate separate bioethics committee review for this category of analysis. One hundred and sixty-nine patients were treated with the device at the centre in total, of whom 128 were treated within the study period; of these, fifty-six consecutive patients with duplex-confirmed isolated ASV insufficiency were included. Inclusion criteria required: (1) symptomatic or clinically significant ASV reflux confirmed on colour duplex ultrasound (DUS), with clinical CEAP classification C2–C4; (2) absence of concurrent GSV or SSV trunk incompetence requiring simultaneous treatment; (3) no use of V-block or endovascular sealing devices at the saphenofemoral junction. Patients with active deep vein thrombosis, known allergy to polidocanol, or severe peripheral arterial disease were excluded.

The study was conducted in accordance with the ethical principles of the Declaration of Helsinki. Follow-up assessments were conducted at 1, 2, 6, and 12 months post-procedure according to the institutional protocol, incorporating clinical evaluation and duplex ultrasound. Baseline demographic, clinical and duplex variables—age, sex, treated side, clinical CEAP class, previous treatment of the ipsilateral limb, maximum ASV diameter within the segment to be treated, reflux duration, and competence of the saphenofemoral junction and of the deep venous system—were retrieved from the institutional database and are presented in Table 1; the procedural protocol is summarised separately in Table 2. Occlusion was defined as absence of detectable flow with non-compressible hyperechoic luminal content along the whole treated segment; primary failure denotes absence of occlusion at the 1-month examination and recanalisation denotes restoration of flow in a previously occluded segment. No venous severity score, pain score, or quality-of-life instrument formed part of the institutional protocol during the study period.

### 2.2. The Sclerosafe Technique

All procedures were performed in an outpatient setting under local anaesthesia, without the need for tumescent infiltration. Under continuous ultrasound guidance, a Sclerosafe catheter (VVT Medical, Kefar Sava, Israel) was introduced into the target ASV segment using an aseptic technique. Access was obtained by direct percutaneous puncture of the target vein itself at the lowest point of reflux identified on duplex ultrasound, most commonly in the proximal half of the thigh, after infiltration of 2 mL of 1% lignocaine at the puncture site only. No introducer sheath was required by the technique: the catheter was advanced directly through the puncture and positioned with its tip 2 cm distal to the confluence of the ASV with the common femoral vein, confirmed on transverse and longitudinal ultrasound in every case. A single puncture was sufficient in all patients, and neither additional access nor catheter exchange was needed, irrespective of the length of the refluxing segment. The length of the catheter does not constrain the length of vein that can be treated, because the relationship between the two is not one-to-one: the foam delivered under aspiration distributes along the segment as the catheter is withdrawn, so segments longer than the catheter were treated from the same single puncture. The dual-lumen design of the catheter enables simultaneous aspiration of blood from the treated venous segment via one port, while the sclerosant is delivered through the second port. This aspiration mechanism is intended to maximise displacement of intraluminal blood prior to and during sclerosant delivery, thereby enhancing sclerosant-to-endothelium contact—the primary determinant of obliteration efficacy [7,8]. Aspiration was maintained continuously while the catheter was withdrawn from the tip position towards the puncture site, so that the treated segment corresponds to the length of vein between these two points. The volume of blood aspirated was not measured. Aspiration removes foam together with blood once delivery has begun, so the content of the aspiration syringe does not represent displaced blood alone and cannot be used to quantify blood displacement. Adequacy of displacement was therefore judged sonographically, as replacement of intraluminal blood echoes by homogeneous echogenic foam, and was not quantified.

Polidocanol foam at a concentration of 2% was prepared using the Tessari double-syringe technique at a gas-to-liquid ratio of 4:1, consistent with European guidelines for sclerotherapy [8]. A volume of 5 mL of foam was administered per procedure in every patient, irrespective of vein diameter; this volume corresponds to the capacity of the delivery syringe, which was not refilled during the procedure, and lies within the guideline ceiling of 10 mL of foam per session [8]. All 56 limbs were treated in a single session. The treated venous segment ranged from 8 to 17 cm in length (mean 10 cm), with baseline ASV diameters between 4 and 12 mm (mean 6 mm). Compression therapy was standardised for the whole cohort and applied immediately after the procedure in accordance with the institutional protocol, identically in every patient. No pharmacological thromboprophylaxis was routinely administered unless individual risk factors indicated otherwise.

### 2.3. Statistical Analysis

Continuous variables are expressed as mean ± standard deviation (SD) or median with interquartile range (IQR), as appropriate. Categorical variables are reported as absolute frequencies and percentages. Two denominators are reported and are not mixed: the number evaluable at each visit (56 minus the cumulative number lost to follow-up), used for crude occlusion rates, and the number at risk (patients still occluded and still under observation), used for the Kaplan–Meier analysis, in which patients leave the risk set once an occlusion failure has occurred and are censored at the last visit attended. Confidence intervals for the survival function were obtained with the Greenwood formula, and the upper confidence limit for adverse events with no observed occurrence by the rule of three. No inferential comparisons between groups were performed, as the study comprised a single-arm cohort, and no subgroup analysis by vein diameter or clinical CEAP class was undertaken because the number of events was insufficient to support one. Statistical analyses were performed using SPSS version 27.0 (IBM Corp., Armonk, NY, USA).

## 3. Results

### 3.1. Procedural Outcomes

All 56 procedures were completed as planned. Technical success—defined as successful catheter placement and sclerosant delivery with visible echogenic foam distribution within the target segment on intraoperative ultrasound—was achieved in 100% of cases. No procedure required conversion to an alternative modality or intraoperative intervention. All patients tolerated the procedure under local anaesthesia without analgesic supplementation.

### 3.2. Follow-Up and Efficacy

**Figure 1:**
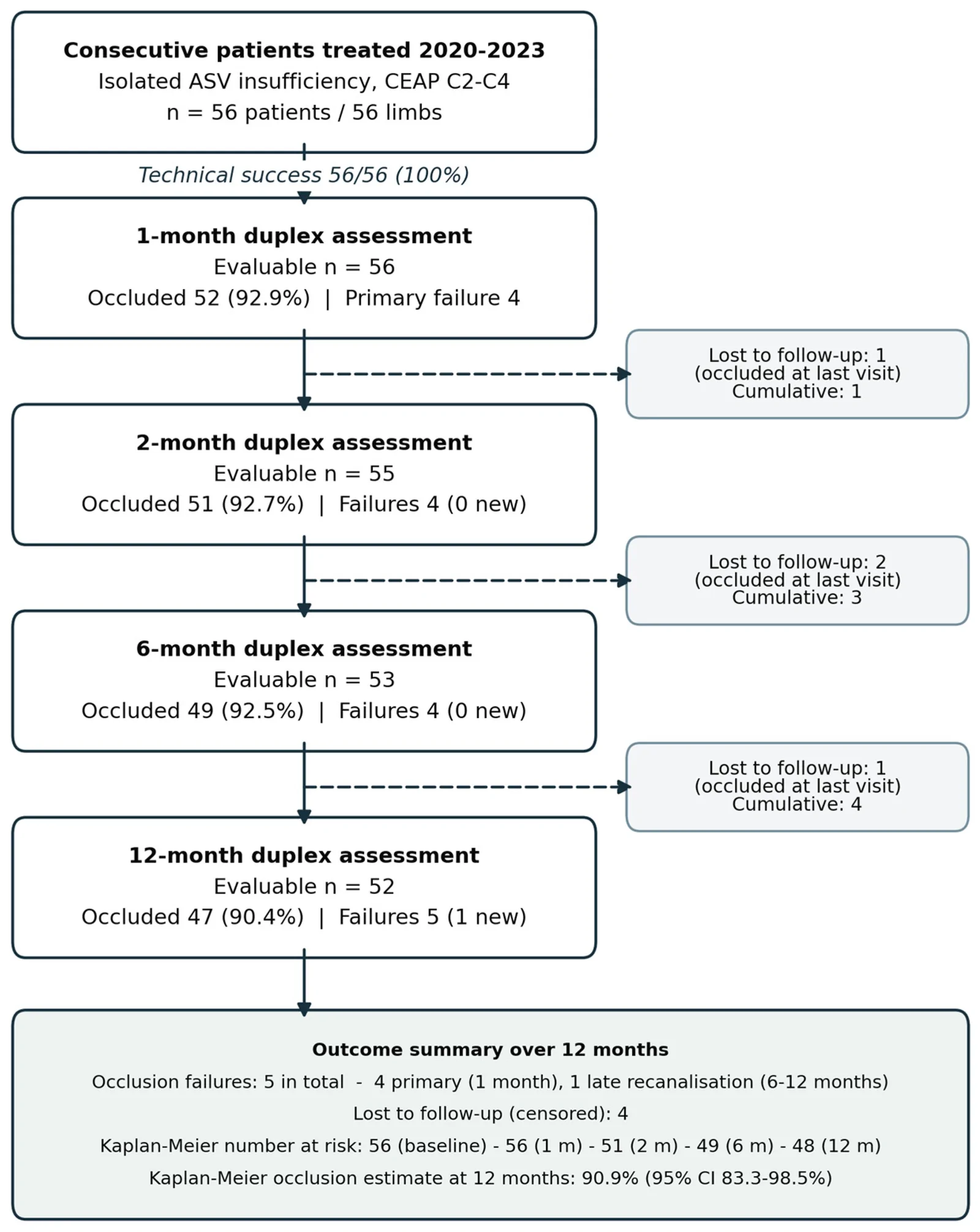
Patient flow through the study. Each box gives the number evaluable, the number and proportion occluded, and the cumulative number of occlusion failures, with new failures shown in parentheses. Losses to follow-up are shown to the right and are cumulative, so the number evaluable decreases monotonically (56, 55, 53, 52); all four patients lost to follow-up had an occluded segment at their last examination. Kaplan–Meier numbers at risk (56, 56, 51, 49, 48) exclude patients in whom a failure has already occurred and are therefore lower than the numbers evaluable (Table 2, Figure 2).

Duplex ultrasound assessment at one month demonstrated complete occlusion of the treated ASV segment (absence of detectable flow and hyperechoic luminal content) in 52 of 56 patients (92.9%). At 2 months, occlusion was confirmed in 51 of 55 evaluable patients (92.7%), and one patient was lost to follow-up. At 6 months, 49 of 53 evaluable patients (92.5%) demonstrated sustained obliteration, with two further losses to follow-up. At 12 months, 47 of 52 evaluable patients (90.4%) showed continued occlusion; four cumulative losses to follow-up were recorded over the study period. All four patients lost to follow-up had an occluded treated segment at their last available examination. The flow of patients through the study is shown in Figure 1.

**Figure 2:**
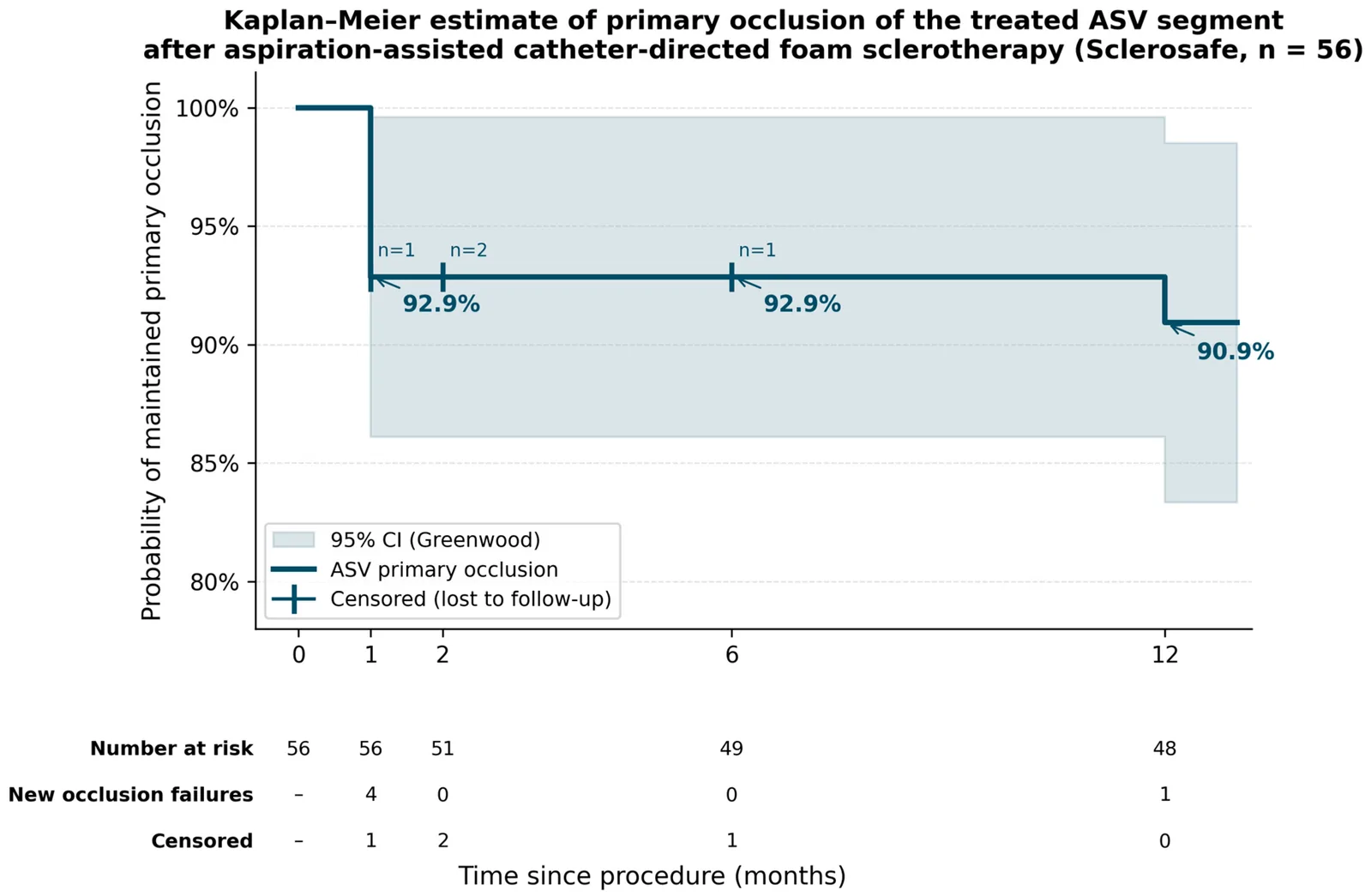
Kaplan–Meier estimate of primary occlusion probability following aspiration-assisted catheter-directed foam sclerotherapy using the Sclerosafe device (VVT Medical, Kefar Sava, Israel) for anterior saphenous vein (ASV) insufficiency (n = 56). Censored observations (patients lost to follow-up) are indicated by vertical tick marks, annotated with the number censored at each timepoint. The table below the x-axis gives, at each timepoint, the number at risk, the number of new occlusion failures, and the number of observations censored at that visit; that is, patients whose last attended examination was that one. Such a patient appears in Table 3 as lost to follow-up at the next scheduled visit, so the censoring counts here (1, 2 and 1 at 1, 2 and 6 months) correspond to the cumulative losses in Table 3 (1, 3 and 4 at 2, 6 and 12 months). The number at risk (56, 56, 51, 49, 48) decreases monotonically and differs by design from the number evaluable in Table 3 (56, 55, 53, 52), which retains patients with a previously documented failure. Shaded band: 95% confidence intervals (Greenwood formula). KM estimates: S(1 m) = 92.9% (95% CI 86.1–99.6%); S(6 m) = 92.9% (no new events between 1 and 6 months); S(12 m) = 90.9% (95% CI 83.3–98.5%). Occlusion failures: t = 1 month, n = 4; t = 12 months, n = 1.

The 4 patients without occlusion at one month were all re-assessed at 2 and 6 months, and no further recanalisation event was identified in this interval (49 of 53 evaluable patients, 92.5%). One additional recanalisation was documented between 6 and 12 months (47 of 52 evaluable patients, 90.4%), yielding 5 occlusion failures in total. The crude occlusion rate therefore declines slightly at each successive timepoint and does not rise, as it would under a fixed set of events. Kaplan–Meier estimates of primary occlusion probability, accounting for 4 censored observations and with numbers at risk of 56, 51, 49 and 48 at 1, 2, 6 and 12 months, were 92.9% at 1 month (95% CI 86.1–99.6%), 92.9% at 6 months (95% CI 86.1–99.6%; no new events), and 90.9% at 12 months (95% CI 83.3–98.5%) (Figure 2).

No cases of deep vein thrombosis, pulmonary embolism, or clinically significant neurological complications were observed during the study period; with no such event in 56 patients, the one-sided 97.5% upper confidence limit for their true incidence is 6.4%. Minor adverse events included transient post-procedural bruising (11 patients, 19.6%), localised tenderness (6 patients, 10.7%), and superficial skin pigmentation (8 patients, 14.3%); all resolved without specific intervention.

The flow of patients through the study is shown in Figure 1 and the Kaplan–Meier estimate in Figure 2. Occlusion and follow-up data by timepoint are presented in Table 3, baseline characteristics are presented in Table 1, and the procedural protocol is presented in Table 2.

## 4. Discussion

This retrospective series demonstrates that aspiration-assisted catheter-directed foam sclerotherapy using the Sclerosafe device is technically feasible, safe, and anatomically effective for the treatment of ASV insufficiency; no venous severity or patient-reported outcome was recorded, so clinical effectiveness cannot be inferred from these data. The absence of major adverse events and the high rate of technical success across a range of vessel diameters (4–12 mm) and segment lengths (8–17 cm) support the versatility of this approach.

In contextualising these outcomes against thermal alternatives, published series of endovenous laser ablation (EVLA) for the ASV report occlusion rates of 90–98% at 12 months, and radiofrequency ablation (RFA) series yield comparable figures [9]. These results, however, are achieved at the cost of perivenous tumescent anaesthesia, which increases procedural complexity and patient discomfort, and carries a recognised risk of cutaneous nerve irritation given the superficial and variable course of the ASV. The Sclerosafe approach yields 12-month occlusion rates approximating 90%, without tumescent infiltration, in an entirely outpatient setting—a trade-off that may be clinically advantageous in patients with superficial or anatomically tortuous segments where thermal access is technically demanding. This comparison is indirect: the thermal figures derive from separate cohorts with different inclusion criteria and outcome definitions, and the present study had no control arm, so no inference of equivalence is intended.

The broader context for these findings is the recognised evolution of non-thermal, non-tumescent (NTNT) techniques following a decade of routine clinical application. As experience has accumulated, limitations have become apparent—particularly regarding foam volume control, the risk of visual disturbance, and the challenge of treating larger or more tortuous venous segments [5]. The Sclerosafe device targets one of the fundamental determinants of sclerotherapy efficacy: the quality of sclerosant-to-endothelium contact. By evacuating residual blood from the vessel lumen during injection, the system seeks to replicate within a sclerotherapy framework the favourable haemodynamic conditions that thermal devices achieve through direct energy-mediated vascular sealing.

The evidence base for CDFS, relative to conventional injection-based foam sclerotherapy, has been consistently favourable in comparative studies. Published meta-analytic data encompassing 3689 patients document a meaningful difference in three-year occlusion rates: 82.4% for catheter-directed delivery versus 62.9% for standard ultrasound-guided injection [6]. The clinical utility of catheter-directed techniques in anatomically complex cases—including recurrent and post-surgical reflux—has been corroborated by Leiva Hernando et al. [10]. The Sclerosafe device represents an evolution of CDFS methodology through the addition of an aspiration mechanism intended to further improve the haemodynamic conditions for sclerosant action; however, the absence of head-to-head comparisons with other CDFS platforms constitutes a recognised evidential gap. A prospective observational series of the same device in great and small saphenous disease reported high early occlusion at 90 days [11], albeit not in the ASV and not beyond three months, and the other non-thermal non-tumescent modalities have been reviewed elsewhere [5]. Differences between such series in the definition of occlusion, in follow-up schedule and in case mix, together with the under-representation of the ASV in all of them, mean that cross-study figures provide context rather than evidence of equivalence.

Thirty-one of the 56 limbs (55.4%) had undergone previous ipsilateral saphenectomy or GSV ablation, and all 56 had an incompetent saphenofemoral junction. This pattern should not be read as recurrent great saphenous reflux or as failure of the earlier procedure. The ASV is an independent truncal vein with its own saphenous compartment and its own drainage into the saphenofemoral junction [2,3]; its incompetence in a limb whose GSV has already been removed or ablated is therefore new varicose disease arising in a separate trunk. In the terminology of the REVAS consensus and of VEIN-TERM, such veins fall under the umbrella of varices present after intervention but are classified as new varices attributable to disease progression rather than as residual or truly recurrent veins [12,13]. The distinction is not merely semantic: a newly incompetent independent trunk requires ablation in its own right, and the present series indicates that this can be achieved in a previously operated limb without tumescent anaesthesia and without a further open procedure.

The applicability of Sclerosafe to the ASV is particularly notable. Thermal ablation of the ASV is technically demanding owing to its superficial course, variable anatomy, and proximity to cutaneous nerves [2,3]. The avoidance of tumescent anaesthesia and the ability to treat segments within the supraaponeurotic plane expands the procedural accessibility of this technique. The observed mean vessel diameter of 6 mm aligns with the recognised upper boundary for effective sclerotherapy [8]. For vessels exceeding this threshold, alternative or hybrid strategies may warrant consideration.

The relationship between vein diameter and outcome is of practical interest, since the conventional upper limit for reliable sclerotherapy is of the order of 6 mm [8] whereas diameters here extended to 12 mm. The present cohort cannot address this question. With 56 limbs and 5 occlusion failures the number of events is far too small to estimate the effect of diameter on the probability of occlusion with any useful precision, and the same applies to clinical CEAP class; no subgroup analysis was therefore performed and none is reported. The fixed dose compounds the difficulty: because 5 mL of foam was delivered irrespective of diameter, the dose per unit of endothelial surface falls as diameter rises, so diameter and dose cannot be separated in a series of this size. Establishing whether diameter influences outcome, and whether diameter-adjusted dosing improves it, requires a prospective cohort powered for that purpose.

The European guidelines for sclerotherapy in chronic venous disorders recommend ultrasound-guided foam sclerotherapy as an effective treatment for saphenous and accessory vein insufficiency, with catheter-assisted modifications endorsed for enhanced efficacy [8]. The ESVS 2022 Clinical Practice Guidelines further reinforce the role of minimally invasive, endovenous techniques across the spectrum of superficial venous disease, including accessory vein pathology [4]. The present findings are consistent with this framework, supporting Sclerosafe as a clinically rational addition to the armamentarium for ASV insufficiency.

Three further caveats deserve emphasis. First, although no deep vein thrombosis, pulmonary embolism or neurological event occurred, 56 patients provide limited precision: the one-sided 97.5% upper confidence limit for the true incidence of such events is 6.4%, so complications with a background incidence of the order of 1% would not be expected to appear reliably, and registry-scale data are required to exclude them. Second, follow-up did not extend beyond 12 months, whereas recanalisation after randomised data comparing foam sclerotherapy with thermal ablation already shows lower occlusion for foam at one year [9], and durability beyond that point cannot be assumed from the present series; surveillance of this cohort at 24 and 36 months is planned and will be reported separately. Third, no patient-reported outcome was captured, and a venous severity score, a periprocedural pain rating and a disease-specific quality-of-life instrument should be incorporated in prospective work. Occlusion failure in this series was managed within the same non-thermal framework, predominantly by repeat ultrasound-guided foam sclerotherapy, which is straightforward and repeatable without tumescent anaesthesia.

This study is subject to several limitations. The retrospective design and absence of a control group preclude definitive conclusions regarding comparative efficacy relative to thermal or pharmacomechanical techniques. No clinical outcome measures were collected, so the effectiveness reported here is anatomical rather than clinical. The cohort size, while sufficient for a preliminary analysis, limits statistical power for subgroup analyses and leaves the 12-month estimate imprecise (95% CI 83.3–98.5%, 5 events). Duplex assessment was performed by the treating team rather than by blinded independent observers, and although all four patients lost to follow-up were occluded at their last examination, the Kaplan–Meier estimate assumes that censoring is uninformative. Detailed long-term follow-up data extending beyond 12 months are not yet available. These findings should therefore be interpreted as hypothesis-generating, providing the rationale and preliminary evidence to support appropriately powered prospective trials.

## 5. Conclusions

Aspiration-assisted catheter-directed foam sclerotherapy using the Sclerosafe device demonstrates promising safety and anatomical efficacy for the treatment of anterior saphenous vein insufficiency, with a 12-month Kaplan–Meier primary occlusion estimate of 90.9% (95% CI 83.3–98.5%) in this retrospective series of 56 patients. The technique requires no tumescent anaesthesia, is applicable across a clinically relevant range of vessel diameters and segment lengths, and was delivered without major adverse events in an outpatient setting. Because clinical severity and patient-reported outcomes were not recorded, and there was no control group, the evidence presented is anatomical, and these findings provide a basis for further prospective evaluation. Randomised or registry-based comparative studies will be required to define the place of this technique within the evidence hierarchy for ASV treatment.

## Figures and Tables

**Table 1:** Baseline patient, clinical, and duplex characteristics (n = 56).

Characteristic	Value
Patients (limbs) treated, n	56 (56)
Age, years, median (range)	48 (30–79)
Female sex, n (%)	40 (71.4)
Male sex, n (%)	16 (28.6)
Clinical CEAP class C2–C4, n (%)	56 (100)
Previous ipsilateral saphenectomy or GSV ablation, n (%)	31 (55.4)
Incompetent saphenofemoral junction, n (%)	56 (100)
Maximum ASV diameter in treated segment, mm, mean (range)	6 (4–12)
Length of ASV segment treated, cm, mean (range)	10 (8–17)

ASV: anterior saphenous vein; CEAP: Clinical–Etiological–Anatomical–Pathophysiological classification; GSV: great saphenous vein. Duplex measurements were obtained in the standing position. All limbs were of clinical CEAP class C2–C4 according to the inclusion criteria.

**Table 2:** Procedural protocol (n = 56).

Protocol Item	Specification
Setting and anaesthesia	Outpatient day case; 2 mL of 1% lignocaine at the puncture site only; no tumescent infiltration
Access	Direct percutaneous ultrasound-guided puncture of the ASV at the lowest point of reflux, most commonly in the proximal half of the thigh; single puncture; no introducer sheath
Catheter and tip position	Sclerosafe dual-lumen catheter advanced directly through the puncture; tip 2 cm distal to the confluence of the ASV with the common femoral vein, confirmed on ultrasound in all cases
Treated length	Not limited by catheter length; the relationship is not one-to-one, and segments longer than the catheter were treated from the same single puncture
Sclerosant and volume	Polidocanol 2% foam, Tessari technique, 4:1 gas-to-liquid; 5 mL per procedure, corresponding to the capacity of the delivery syringe, which was not refilled
Delivery	Continuous aspiration with simultaneous foam delivery during withdrawal from the tip position to the puncture site
Volume of blood aspirated	Not measured; foam is aspirated together with blood, so the aspirate does not represent displaced blood alone
Endpoint of delivery	Sonographic replacement of intraluminal blood echoes by homogeneous echogenic foam (qualitative)
Sessions per patient	1 in all 56 patients
Compression	Standardised across the cohort; applied immediately after the procedure according to the institutional protocol
Thromboprophylaxis	Not routine; individualised by risk factors
Follow-up	Clinical examination and duplex ultrasound at 1, 2, 6 and 12 months

ASV: anterior saphenous vein.

**Table 3:** Occlusion and follow-up by timepoint (n = 56).

Measure	1 Month	2 Months	6 Months	12 Months
Evaluable, n ^1^	56	55	53	52
Occluded, n	52	51	49	47
Occlusion rate, %	92.9	92.7	92.5	90.4
New occlusion failures, n ^2^	4	0	0	1
Cumulative occlusion failures, n	4	4	4	5
Lost to follow-up, cumulative, n	0	1	3	4
At risk, Kaplan–Meier, n ^3^	56	51	49	48

^1^ Patients with a duplex examination at that visit (56 minus the cumulative number lost to follow-up); patients with a previously documented failure who continued to attend remain in this denominator. ^2^ Failures first identified at that visit: primary failure at 1 month, recanalisation thereafter; the cumulative row gives all failures up to and including that visit. ^3^ Number at risk in the Kaplan–Meier analysis (Figure 2): patients still occluded and still under observation, who leave the risk set after a failure and are censored at the last visit attended. Both the evaluable and at-risk rows decrease monotonically. All 4 patients lost to follow-up had an occluded segment at their last examination.

## Data Availability

The data presented in this study are available from the corresponding author upon reasonable request. The data are not publicly available because they were collected retrospectively from medical records and contain sensitive patient information. Public sharing is restricted by patient privacy requirements and institutional ethical regulations.

## References

[1] Fan C.M. (2003). Epidemiology and pathophysiology of varicose veins. Tech. Vasc. Interv. Radiol..

[2] Caggiati A., Bergan J.J., Gloviczki P., Jantet G., Wendell-Smith C.P., Partsch H. (2002). Nomenclature of the veins of the lower limbs: An international interdisciplinary consensus statement. J. Vasc. Surg..

[3] Meissner M., Boyle E., Labropoulos N., Caggiati A., Drgastin R., Doganci S., Gasparis A. (2024). The anterior saphenous vein. Part 1. A position statement endorsed by the American Vein and Lymphatic Society, the American Venous Forum, and the International Union of Phlebology. J. Vasc. Surg. Venous Lymphat. Disord..

[4] De Maeseneer M.G., Kakkos S.K., Aherne T., Baekgaard N., Black S., Blomgren L., Giannoukas A., Gohel M., de Graaf R., Hamel-Desnos C. (2022). Editor’s Choice—ESVS 2022 Clinical Practice Guidelines on the Management of Chronic Venous Disease of the Lower Limbs. Eur. J. Vasc. Endovasc. Surg..

[5] Bootun R., Lane T.R.A., Davies A.H. (2016). The advent of non-thermal, non-tumescent techniques for treatment of varicose veins. Phlebology.

[6] Lim S.Y., Tan J.X.D., D’Cruz R.T., Syn N., Chong T.T., Tang T.Y. (2020). Catheter-directed foam sclerotherapy, an alternative to ultrasound-guided foam sclerotherapy for varicose vein treatment: A systematic review and meta-analysis. Phlebology.

[7] Avrahami M., Silverberg D., Elias S., Kolvenbach R., Shufutinsky N., Sivak G., Tal M., Avrahami R. (2022). Inframalleolar access in endovenous treatment of venous ulcers and C5 disease with nonthermal nontumescent techniques. J. Vasc. Surg. Venous Lymphat. Disord..

[8] Rabe E., Breu F., Cavezzi A., Smith P.C., Frullini A., Gillet J., Guex J., Hamel-Desnos C., Kern P., Partsch B. (2014). European guidelines for sclerotherapy in chronic venous disorders. Phlebology.

[9] Rasmussen L.H., Lawaetz M., Bjoern L., Vennits B., Blemings A., Eklof B. (2011). Randomized clinical trial comparing endovenous laser ablation, radiofrequency ablation, foam sclerotherapy and surgical stripping for great saphenous varicose veins. Br. J. Surg..

[10] Leiva Hernando L., Arroyo Bielsa A., Fletes Lacayo J.C. (2022). Treatment of recurrent symptomatic saphenous trunk reflux with catheter-directed foam sclerotherapy and tumescent anaesthesia. EJVES Vasc. Forum.

[11] Bhuiyan A.S., Sheth M.T. (2026). Efficacy and safety of ScleroSafe catheter-directed sclerotherapy for varicose veins: A prospective observational study. Indian J. Vasc. Endovasc. Surg..

[12] Perrin M.R., Guex J.J., Ruckley C.V., dePalma R.G., Royle J.P., Eklof B., Nicolini P., Jantet G. (2000). Recurrent varices after surgery (REVAS), a consensus document. Cardiovasc. Surg..

[13] Eklöf B., Perrin M., Delis K.T., Rutherford R.B., Gloviczki P. (2009). Updated terminology of chronic venous disorders: The VEIN-TERM transatlantic interdisciplinary consensus document. J. Vasc. Surg..

